# Effect of antenatal multiple micronutrient supplementation on anthropometry and blood pressure in mid-childhood in Nepal: follow-up of a double-blind randomised controlled trial

**DOI:** 10.1016/S2214-109X(14)70314-6

**Published:** 2014-11

**Authors:** Delan Devakumar, Shiva Shankar Chaube, Jonathan C K Wells, Naomi M Saville, Jon G Ayres, Dharma S Manandhar, Anthony Costello, David Osrin

**Affiliations:** a Institute for Global Health, University College London, London, UK; b Institute of Child Health, University College London, London, UK; c Mother and Infant Research Activities, Kathmandu, Nepal; d Institute of Occupational and Environmental Medicine, University of Birmingham, Birmingham, UK

## Abstract

**Background:**

In 2002–04, we did a randomised controlled trial in southern Nepal, and reported that children born to mothers taking multiple micronutrient supplements during pregnancy had a mean birthweight 77 g greater than children born to mothers taking iron and folic acid supplements. Children born to mothers in the study group were a mean 204 g heavier at 2·5 years of age and their systolic blood pressure was a mean 2·5 mm Hg lower than children born to mothers in the control group. We aimed to follow up the same children to mid-childhood (age 8·5 years) to investigate whether these differences would be sustained.

**Methods:**

For this follow-up study, we identified children from the original trial and measured anthropometry, body composition with bioelectrical impedance (with population-specific isotope calibration), blood pressure, and renal dimensions by ultrasound. We documented socioeconomic status, household food security, and air pollution. Main outcomes of the follow-up at 8 years were *Z* scores for weight-for-age, height-for-age, and body-mass index (BMI)-for-age according to WHO Child Growth Standards for children aged 5–19 years, and blood pressure. This study is registered with the International Standard Randomised Controlled Trial register, number ISRCTN88625934.

**Findings:**

Between Sept 21, 2011, and Dec 7, 2012, we assessed 841 children (422 in the control group and 419 in the intervention group). Unadjusted differences (intervention minus control) in *Z* scores were 0·05 for weight-for-age (95% CI −0·09 to 0·19), 0·02 in height-for-age (−0·10 to 0·15), and 0·04 in BMI-for-age (−0·09 to 0·18). We recorded no difference in blood pressure. Adjusted differences were similar for all outcomes.

**Interpretation:**

We recorded no differences in phenotype between children born to mothers who received antenatal multiple micronutrient or iron and folate supplements at age 8·5 years. Our findings did not extend to physiological differences or potential longer-term effects.

**Funding:**

The Wellcome Trust.

## Introduction

Data for the developmental origins of health and disease suggest that early-life experience can have lasting effects on growth and physiology. The mechanisms include an interplay between environment, genes, and hormones, in which epigenetic regulation plays a part. How tractable this interplay is to environmental effects, how long the tractability lasts, and whether it is reversible, remain uncertain.^1^, ^2^, ^3^

Macronutrients and micronutrients are both important for the short-term and long-term health of mothers and children, especially in resource-poor settings in which women can have many deficiencies.^2^, ^4^, ^5^ Many women take iron and folic acid before or during pregnancy, and a supplement containing 15 vitamins and minerals developed by UNICEF, the United Nations University, and WHO has been considered for use in pregnancy.^6^ We previously did a double-blind randomised controlled trial^7^ with pregnant women in which we compared the effects of taking this multiple micronutrient supplement every day in the second and third trimesters of pregnancy with supplements of iron and folic acid. We found an increase of 77 g in birthweight (95% CI 24–130) in the intervention group, with a corresponding 25% relative reduction in low birthweight prevalence.^7^ Ours was one of several trials of this supplement,^8^, ^9^, ^10^, ^11^, ^12^, ^13^, ^14^, ^15^ and findings of meta-analyses of these and similar trials have shown an increase in birthweight of between 22 g and 54 g, a reduction in low birthweight and small-for-gestational age, but no other changes in anthropometry, gestation, or mortality.^16^, ^17^, ^18^, ^19^, ^20^

Children born in our trial were followed up at age 2·5 years. The multiple micronutrient group were a mean 204 g (95% CI 27–381) heavier, with small increases in body circumferences and a mean systolic blood pressure 2·5 mm Hg lower (0·5–4·6) than those in the control group.^21^ We have now followed up children at 8 years of age, hypothesising that the differences in weight and blood pressure recorded at 2·5 years would be maintained in relative terms into mid-childhood. We repeated the anthropometry and blood pressure measurements, and also examined body composition.

## Methods

### Study design and participants

The study was based in Dhanusha district in the Central Terai of Nepal, close to the Indian border. Nepal is one of the world's poorest countries. Gross domestic product is US$35·81 billion and per person income is $1160 (at purchasing power parity), which places Nepal 103rd in terms of wealth.^22^, ^23^ The Human Development Index is 0·458, ranked 157th worldwide.^23^

The trial has been described previously.^7^ 1200 women attending Janakpur Zonal Hospital for antenatal care were randomly allocated to receive either a multivitamin supplement (containing 800 μg vitamin A, 10 mg vitamin E, 5 μg vitamin D, 1·4 mg vitamin B1, 1·4 mg vitamin B2, 18 mg niacin, 1·9 mg vitamin B6, 2·6 μg vitamin B12, 400 μg folic acid, 70 mg vitamin C, 30 mg iron, 15 mg zinc, 2 mg copper, 65 μg selenium, and 150 μg iodine) or a control supplement of 60 mg iron and 400 μg folic acid.^6^ Supplements were taken every day from 12 to 20 weeks' gestation (average 15·9 weeks) until delivery, and women were assessed every 2 weeks. Exclusion criteria included multiple pregnancies (ie, twins or more), fetal abnormalities on obstetric ultrasound, and maternal illness that could compromise the outcome of the pregnancy. 1069 mothers and infants completed the trial and were seen by the study team at birth and at 1 month of age. Participants, their families, and research staff were masked to trial allocation.

The follow-up study was approved by the UCL research ethics board (reference 2744/001) and the Nepal Health Research Council (reference 51/2011). Parents or guardians of children gave signed informed consent in their local language.

### Procedures

We tried to find children from the original trial with location data from previous follow-up. We travelled anywhere in Dhanusha and adjoining districts, and also assessed 14 children who had moved to Kathmandu or the town of Hetauda.

A questionnaire was developed in Maithili, Nepali, and English, back-translated to ensure equivalence, piloted in the local population, and adapted before use. Modules covered socioeconomic status, food security, and parental recall of diarrhoeal and respiratory illnesses in the past week and year. We assessed food security with the Household Food Insecurity Access Scale (HFIAS)^24^ and the Household Dietary Diversity Score (HDDS),^25^ which have been used in other research in the specialty.^26^ We used the HFIAS to work out the level of food insecurity experienced by a household during the past 30 days, in terms of anxiety about food access, quality, and quantity of food. We used the HDDS to examine the breadth of the child's diet in the preceding week.

For anthropometric measurements, we followed guidelines by UCL Institute of Child Health (based on an anthropometric standardisation reference manual)^27^ and WHO.^28^ We attempted to minimise biological variation by taking measurements at a similar time of day. We did duplicate measures of standing height with a Leicester stadiometer (Invicta Plastics, UK), accurate to 1 mm. The child's footwear and hair accessories were removed and they were positioned with their head and back touching the stadiometer, with knees extended and feet together, heels touching the base of the stadiometer, and head in the Frankfort plane. For sitting height, the child was seated on a custom-made stool with the base of the spine touching the stadiometer and head in the Frankfort plane.

Weight and body composition were measured with a Tanita BC-418 scale (Tanita, Japan) accurate to 0·1 kg. Children were given a standard set of clothes (underwear, vest, and sarong) weighing 200 g, which they wore instead of their own. Before assessment, the child was asked to pass urine. Body composition was estimated with a bioelectrical impedance analysis, with a population-specific calibration study that used isotope dilution. Bioelectrical impedance analysis uses a calibration equation to convert electrical impedance to an estimate of total body water, and to in turn generate an estimate of lean mass, relying on the assumption that lean mass and fat mass have different and consistent impedance to current. We did isotope calibration by measuring total body water with 0·06 mg/kg deuterium oxide with 100 children aged 7–9 years. We converted total body water to lean mass by use of age-specific and sex-specific hydration values.^29^ We selectively sampled children of different weights to produce as accurate a prediction equation as possible: lean mass (kg)=1·95 + (0·68 × height [cm]^2^ / impedance [Ω]) + (0·21 × weight [kg]) – (0·36 × male sex); *R*^2^=0·93, root mean squared error=0·95 kg.

We measured body circumferences in duplicate with Seca 201 tapes (Seca, Germany) accurate to 1 mm with the child standing in the anatomical position. Maximum horizontal head circumference was measured around the forehead and occiput, under the hair if needed, in the Frankfort plane. Chest circumference was measured at the nipple line at the end of normal expiration. To measure the waist circumference, the child was asked to bend from one side to the other and the points of flexure were marked. We placed the measuring tape around the abdomen at these points and read when the child was relaxed at the end of normal expiration. Hip circumference was measured at the widest girth, mid-upper arm circumference midway between the olecranon process, and the tip of the acromion and upper leg circumference midway between the greater trochanter and the lateral femoral epicondyle. We calibrated instruments every 2 weeks.

Left biceps, triceps, subscapular and supra-iliac skinfold thicknesses were measured in triplicate with a Harpenden calliper (Assist Creative Resource, UK), accurate to 0·2 mm and calibrated against a Vernier calliper. We used an Omron M6 electronic blood pressure monitor (Omron Healthcare, Japan) with a paediatric cuff, or adult cuff if needed. Measurements followed Great Ormond Street Hospital for Children guidelines.^30^ Blood pressure was recorded after the child had been seated for at least 1 min with legs uncrossed. The child was told to relax with head back and right arm on the armrest at the level of the sternum. Two readings were taken, with the cuff deflated fully and 1 min between them, and the lowest value was recorded.

Measurements of kidney size were made to examine a potential mediator of blood pressure difference. Ultrasound measurements were made by a clinician trained in ultrasonography with an Aloka SDD-500 (Aloka, Japan) with a 2–8 MHz convex probe (Aloka, Japan), accurate to 1 mm. We recorded maximum renal length and anteroposterior dimensions, after visualising sinus and parenchyma using predefined landmarks.

Air pollution is a potential environmental confounder that has been associated with abnormal growth, either directly through, for example, lung damage, or indirectly through an increase in morbidity.^31^ Personal exposure to air pollution was estimated with both gravimetric and photometric sampling of respirable particle mass.^32^, ^33^ Briefly, sampling was done in the microenvironments in which children resided (eg, bedroom, kitchen during and outside cooking hours, outdoors, school, and verandah) three times per year. With time activity data from the questionnaire, we calculated a 24 h time-weighted-average. Estimates were restricted to children living in the plains.

Each child was given a T-shirt, refreshments, and a voucher to be seen by a paediatrician outside the research team, with costs of minor acute treatments covered as a gesture of thanks. Guardians were compensated for travel costs.

We entered data into a Base Open Office database (Apache Software foundation) and Microsoft Excel (version 14·3·2). Data files containing personal details were password protected. No analyses or shared data included the names of participants or families. Questionnaires were stored in a locked office with a security guard.

### Statistical analysis

The study was powered at 81% to detect a difference of 0·2 *Z* scores between allocation groups with 400 children in each, at 5% significance level. This corresponded to the 0·2 *Z* score weight difference recorded in the trial and the previous follow-up, and would be roughly 800 g at 8 years of age. Analysis was done in Excel (version 14.3.2), Prism (version 6.0a), and Stata (version 12.1). We made Bland-Altman plots for all repeat measures to look at reproducibility^34^ and outliers identified. We calculated intra-observer and inter-observer technical error of measurement (TEM)^35^ and coefficient of reliability (*R*)^28^, ^36^, ^37^ and relative TEM (TEM%) allowed the comparison of variables.^38^

The WHO Child Growth Standards for children aged 5–19 were applied to generate *Z* scores for weight, height, and BMI for age.^39^ We assessed distributions for normality. The primary analysis compared allocation groups with *t* tests and univariable regression models by intention to treat.

Although allocation was balanced, we adjusted for potential confounders to increase precision. We constructed a causal diagram based on a priori assumptions and putative associations between variables (appendix). Based on this diagram, multivariable linear regression models included covariates describing air pollution (24 h time-weighted-average), dietary diversity (HDDS score), food security (HFIAS score), maternal education (no education, primary school, or secondary and above) and height, household asset score, and residence (urban or rural). Adjustment for maternal height was not deemed essential, but was included in the model to augment information about diet and socioeconomic status. We included covariates for maternal education and residence to offset the potential effects of differential loss to follow-up.^40^ Model assumptions were tested for linearity by plotting residuals against each covariate, for normality by examining a kernel density plot of residuals, for multi-co-linearity by calculating variance inflation factor, and for heteroskedasticity with the Breusch-Pagan test and plotting residuals against predicted values. The multivariable models seemed linear, had normally distributed residuals, and showed no evidence of co-linearity. Outcomes were usually heteroskedastic and robust standard errors were used.^41^

For the main outcomes, we investigated the direct effects of antenatal multiple micronutrient supplementation, not mediated by birth size. We included anthropometric data at birth in multivariable regression models developed as above.

We excluded 34 children with major or chronic illness and looked at the effect of antenatal supplementation on girls and boys separately. Illnesses were self-reported and classified by a member of the research team trained in paediatrics.

We examined conditional growth to see if growth in a given time differed from the growth expected on the basis of previous measurements. The indicative variable was calculated as the standardised residual on the basis of previous *Z* scores using the WHO reference ranges.^39^, ^42^ A positive value represented growth faster than expected and a negative value, slower. Conditional growth was calculated for two timepoints—2·5 years and 8·5 years. We regressed current size on previous measures with the method described by Adair and colleagues.^43^ Conditional height took into account previous height and weight, whereas conditional relative weight accounted for these and current height.

### Role of the funding source

The funder played no part in the study design, data collection, analysis, interpretation of results, writing the report, or the decision to submit for publication. The corresponding author had full access to all the data in the study and had final responsibility for the decision to submit for publication.

## Results

We visited 852 families between Sept 21, 2011, and Dec 7, 2012. One guardian declined consent and three completed questionnaires only. Retention rates were 81% for the control group and 80% for the intervention group. The figure shows the trial profile. Mean ages at follow up were 8·44 years in the intervention group and 8·47 in the control group. Since the previous follow-up, one child had died in the intervention group and two in the control group. Neonatal mortality had been non-significantly greater in the intervention group,^7^ but mortality rates were similar over the entire follow-up period (appendix). Other than for fever (in 81% of children in the past year; appendix), illness rates were low and similar in intervention and control groups. Children lost to follow-up were similar for most indicators to those retained in the study (table 1). They were more likely to be urban residents (p<0·0001) and their mothers to have some education (p<0·0001).

**Figure:**
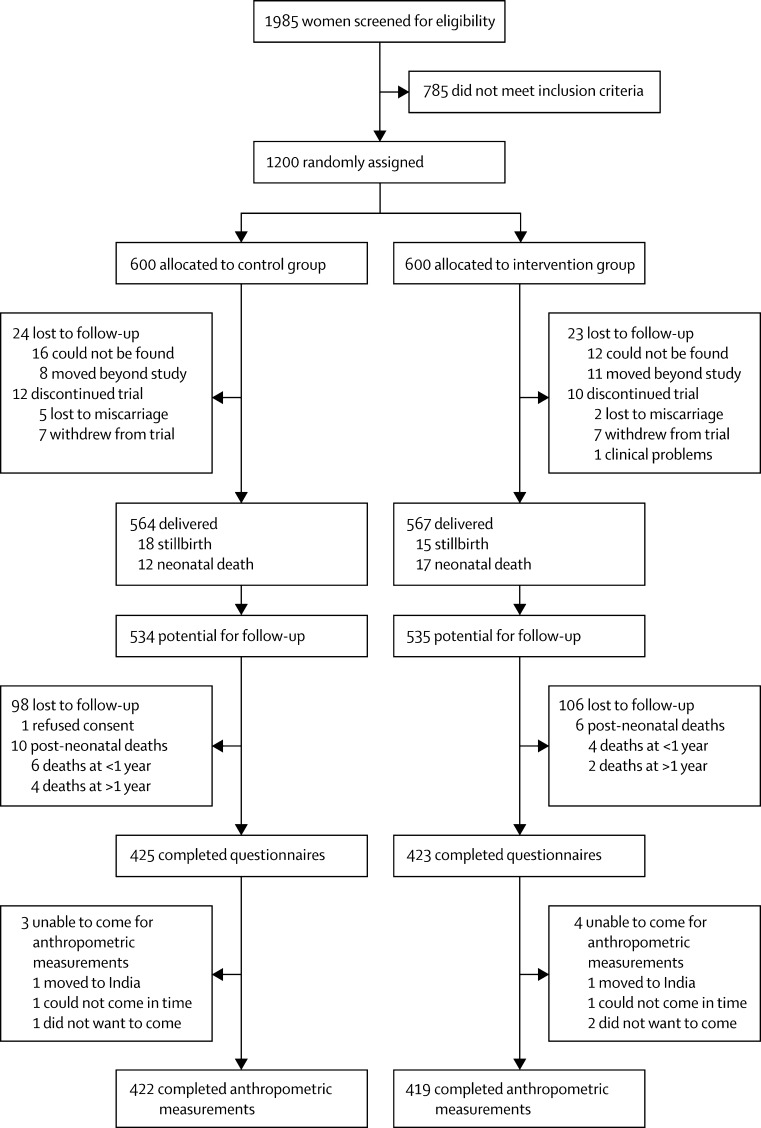
Study profile

**Table 1:** Characteristics of children retained at 8 years and of those lost to follow-up

		**8 year follow-up**		**Lost to follow-up**	
		Control group (n=422)	Intervention group (n=419)	Before end of trial* (n=69)	After end of trial† (n=290)
Residence					
	Urban	199 (47%)	197 (47%)	47 (68%)	184 (64%)
	Rural	223 (53%)	222 (53%)	22 (32%)	106 (37%)
District					
	Dhanusha	348 (83%)	336 (80%)	59 (86%)	245 (85%)
	Mahotari	74 (18%)	79 (19%)	10 (15%)	43 (15%)
	Siraha	0	2 (1%)	0	1 (0%)
	Sarlahi	0	2 (1%)	0	1 (0%)
Mother's age at enrolment					
	<20 years	126 (30%)	130 (31%)	20 (29%)	85 (29%)
	20–29 years	273 (65%)	272 (65%)	44 (64%)	196 (68%)
	≥30 years	23 (6%)	17 (4%)	5 (7%)	9 (3%)
Maternal education					
	None	206 (49%)	210 (50%)	27 (39%)	101 (35%)
	Primary	37 (9%)	33 (8%)	16 (23%)	37 (13%)
	Secondary or higher	179 (42%)	176 (42%)	26 (38%)	152 (52%)
Mean (SD) maternal height mean (cm)		151·0 (6)	150·4 (5)	150·4 (5)	151·0 (6)
Ethnic origin					
	Terai Brahmin or Chhetri	62 (15%)	65 (16%)	13 (19%)	62 (21%)
	Terai Middle Madeshi	296 (70%)	276 (66%)	38 (55%)	176 (61%)
	Terai Janjati or Dalit	10 (2%)	14 (3%)	2 (3%)	3 (1%)
	Hill Brahmin or Chhetri	24 (6%)	23 (6%)	6 (9%)	19 (7%)
	Terai Muslim	23 (6%)	29 (7%)	8 (12%)	17 (6%)
	Janjati (hill)	7 (2%)	11 (3%)	2 (3%)	12 (4%)
	Other	0 (0%)	1 (0%)	0 (0%)	1 (0%)
Main household livelihood					
	No work	49 (12%)	46 (11%)	1 (2%)	34 (12%)
	Farming	72 (17%)	72 (16%)	7 (10%)	34 (12%)
	Salaried	153 (36%)	178 (43%)	34 (49%)	148 (51%)
	Small business	82 (19%)	76 (18%)	19 (28%)	46 (16%)
	Waged labour	53 (13%)	43 (10%)	5 (7%)	18 (6%)
	Student	7 (2%)	3 (1%)	3 (4%)	4 (1%)
	Out of country	6 (1%)	5 (1%)	0 (0%)	6 (2%)
Land ownership					
	0	47 (11%)	44 (11%)	8 (12%)	30 (10%)
	<30 dhur (about 500 m^2^)	290 (69%)	294 (70%)	45 (65%)	196 (68%)
	>30 dhur	85 (21%)	81 (19%)	16 (23%)	64 (22%)
Appliance score					
	Motor vehicle, TV, or refrigerator	217 (51%)	214 (51%)	36 (53%)‡	146 (50%)
	Sewing machine, cassette player, camera, fan, bullock cart, wall clock, radio, iron, or bicycle	147 (35%)	138 (33%)	21 (31%)	95 (33%)
	None of the above	58 (14%)	67 (16%)	11 (16%)	49 (17%)
Parity					
	0	185 (44%)	182 (43%)	33 (48%)	140 (48%)
	1-2	183 (43%)	202 (48%)	28 (41%)	124 (43%)
	≥3	54 (13%)	35 (8%)	8 (12%)	26 (9%)
Preterm (<37 weeks' gestation by ultrasound assessment)		29 (7%)	28 (7%)	‡	41 (14%)
Delivery site					
	Hospital	218 (51·7)	252 (60·1)	‡	176 (60·7)
	Home	194 (46·0)	165 (39·4)	‡	100 (34·5)
	On the way	10 (2·4)	2 (0·5)	‡	14 (4·8)
Child sex and weight					
	Girl	210 (50%)	196 (47%)	‡	152 (54%)‡
	Boy	212 (50%)	223 (53%)	‡	132 (46%)
	Mean (SD) birthweight (kg)	2·74 (0·41)	2·81 (0·43)	‡	2·75 (0·50)

Data are frequency (%) unless otherwise stated.

* 14 women withdrew from the trial, one dropped out with a clinical problem, 19 moved beyond study area, 28 were untraceable, and seven had miscarriages.

† Four refused to attend, two moved beyond study area, two not assessable within follow-up, 204 untraceable, 33 stillbirths, 29 neonatal deaths, and 16 post-neonatal deaths.

‡ Incomplete data at delivery.

Intraobserver TEM% was less than 0·25% (most values <0·1%) and TEM values were less than 0·6 mm, with the exception of skinfold thickness, which was 1–2% (TEM ≤0·1 mm), and renal anteroposterior dimensions at about 5% (TEM <2 mm). With the exception of head, waist, and chest circumferences, interobserver variability was low, with *R* values of 0·93 or higher. Controlling for the effect of observer made little difference and did not change the inferences.

We took anthropometric measurements for 841 children. At 8·5 years of age, roughly 50% of children had low weight-for-age, and a third had low height-for-age and low BMI-for-age (table 2). Weight-for-age scores fell consistently with age, but height-for-age showed some recovery after age 2·5 years. The mean fat mass proportion was 14·2%. Some children were overweight (BMI-for-age >1 SD): 0·5% of girls and 2·3% of boys. We recorded no difference between allocation groups in the main anthropometric outcomes at 8·5 years (table 3). We recorded no difference in systolic and diastolic blood pressure between the groups.

**Table 2:** Anthropometric indices at birth, 2·5 years, and 8·5 years, by allocation

	**Number of children**	**Frequency ≤2 SD (%)**	**Mean *Z* score (95% CI)**	
			Control group	Intervention group
Birth*				
Weight-for-age	1044	186 (18%)	−1·28 (−1·35 to −1·19)	−1·08 (−1·17 to 1·00)
Height-for-age	1016	91 (9%)	−0·41 (−0·52 to −0·30)	−0·34 (−0·45 to −0·24)
BMI-for-age	1002	348 (35%)	−1·63 (−1·74 to −1·52)	−1·42 (−1·53 to −1·31)
**2·5 years**				
Weight-for-age	915	340 (37%)	−1·76 (−1·85 to −1·67)	−1·62 (−1·72 to −1·53)
Height-for-age	915	537 (59%)	−2·29 (−2·39 to −2·19)	−2·20 (−2·31 to −2·10)
BMI-for-age	915	54 (6%)	−0·40 (−0·50 to −0·30)	−0·29 (−0·39 to −0·18)
**8·5 years**				
Weight-for-age	841	444 (53%)	−2·08 (−2·18 to −1·98)	−2·03 (−2·13 to −1·93)
Height-for-age	841	242 (29%)	−1·51 (−1·59 to −1·42)	−1·48 (−1·57 to −1·39)
BMI-for-age	841	293 (35%)	−1·67 (−1·76 to −1·58)	−1·63 (−1·72 to −1·53)

BMI=body-mass index.

* Excluding birth data ≤4.5 and >3 *Z* scores.

**Table 3:** Child anthropometric indices by allocation, showing mean values, unadjusted, and adjusted differences at 8·5 years

		**Control group**	**Intervention group**	**Unadjusted difference (95% CI)**	Adjusted difference*(95% CI)	**Adjusted difference restricted to children without major or chronic illness (95% CI)**
Weight (kg)						
	Weight	20·04 (3·31)	20·14 (3·35)	0·10 (−0·35 to 0·55)	0·30 (−0·08 to 0·67)	0·25 (−0·12 to 0·63)
	Lean mass	17·34 (2·44)	17·30 (2·49)	−0·05 (−0·43 to 0·34)	0·10 (−0·23 to 0·43)	0·08 (−0·25 to 0·42)
	Fat mass	3·01 (1·6)	2·94 (1·58)	−0·07 (−0·32 to 0·18)	0·02 (−0·21 to 0·25)	0·05 (−0·18 to 0·27)
Height (cm)						
	Standing	120·72 (5·91)	120·73 (6·06)	0·00 (−0·81 to 0·81)	0·35 (−0·31 to 1·01)	0·27 (−0·38 to 0·93)
	Trunk length	64·14 (2·94)	64·16 (2·96)	0·02 (−0·38 to 0·42)	0·14 (−0·20 to 0·48)	0·07 (−0·28 to 0·41)
Anthropometric scores						
	Weight-for-age	−2·08 (1·01)	−2·03 (1·06)	0·05 (−0·09 to 0·19)	0·09 (−0·03 to 0·22)	0·07 (−0·06 to 0·19)
	Height-for-age	−1·51 (0·92)	−1·48 (0·96)	0·02 (−0·10 to 0·15)	0·06 (−0·05 to 0·17)	0·05 (−0·06 to 0·16)
	BMI-for-age	−1·67 (0·96)	−1·63 (0·98)	0·04 (−0·09 to 0·18)	0·07 (−0·06 to 0·20)	0·05 (−0·08 to 0·17)
Skinfold thickness (mm)						
	Triceps	7·39 (2·56)	7·36 (2·41)	−0·03 (−0·37 to 0·31)	0·07 (−0·24 to 0·38)	0·10 (−0·20 to 0·37)
	Biceps	3·95 (1·34)	3·95 (1·40)	−0·01 (−0·19 to 0·12)	0·06 (−0·11 to 0·23)	0·08 (−0·10 to 0·25)
	Subscapular	4·91 (1·29)	4·93 (1·51)	0·01 (−0·18 to 0·20)	0·06 (−0·12 to 0·24)	0·07 (−0·11 to 0·26)
	Supra–iliac	5·76 (2·54)	5·57 (2·35)	−0·19 (−0·52 to 0·14)	−0·11 (−0·42 to 0·21)	−0·06 (−0·36 to 0·25)
Body circumference (cm)						
	Head	49·19 (1·48)	49·37 (1·47)	0·18 (−0·02 to 0·38)	0·19 (0·02 to 0·37)	0·15 (−0·02 to 0·32)
	Chest	55·59 (3·39)	55·74 (3·64)	0·15 (−0·33 to 0·63)	0·28 (−0·14 to 0·71)	0·27 (−0·16 to 0·70)
	Waist	49·01 (3·76)	49·20 (3·96)	0·19 (−0·33 to 0·71)	0·29 (−0·19 to 0·77)	0·23 (−0·25 to 0·71)
	Hip	57·30 (4·00)	57·36 (4·11)	0·07 (−0·48 to 0·61)	0·33 (−0·14 to 0·81)	0·26 (−0·22 to 0·73)
	Upper leg	31·11 (2·91)	31·21 (2·91)	0·10 (−0·30 to 0·49)	0·26 (−0·09 to 0·61)	0·21 (−0·14 to 0·56)
	Mid–upper arm	15·94 (1·40)	15·99 (1·38)	0·04 (−0·15 to 0·23)	0·11 (−0·06 to 0·27)	0·10 (−0·07 to 0·26)
Renal dimension (cm)						
	Right length	7·90 (0·55)	7·89 (0·57)	−0·01 (−0·08 to 0·07)	0·00 (−0·07 to 0·07)	−0·01 (−0·08 to 0·06)
	Right anteroposterior distance	2·98 (0·26)	3·00 (0·28)	0·02 (−0·01 to 0·06)	0·02 (−0·01 to 0·06)	0·02 (−0·02 to 0·06)
	Left length	8·25 (0·57)	8·22 (0·58)	−0·03 (−0·11 to 0·05)	−0·03 (−0·10 to 0·05)	−0·05 (−0·12 to 0·03)
	Left anteroposterior distance	3·29 (0·32)	3·30 (0·32)	0·02 (−0·03 to 0·06)	0·02 (−0·03 to 0·06)	0·01 (−0·03 to 0·06)
Blood pressure (mm Hg)						
	Systolic	98·06 (7·14)	98·08 (8·10)	0·02 (−1·02 to 1·05)	−0·06 (−1·10 to 0·98)	−0·20 (−1·23 to 0·83)
	Diastolic	61·16 (7·36)	61·29 (8·27)	0·13 (−0·93 to 1·19)	0·19 (−0·87 to 1·25)	0·15 (−0·92 to 1·22)

Data are mean (SD) unless otherwise stated.

* Multivariable regression models included variables describing air pollution, dietary diversity, food security, maternal education and height, household asset score, and residence, by use of robust standard errors. Age and sex were included if not intrinsic to *Z* score.

Multivariable regression models tended to increase the effect size, but the results did not reach statistical significance. The only outcome that did was head circumference (table 3). Table 4 shows stratification by sex; we recorded no differences and no evidence of interaction (p=0·24).

**Table 4:** Child anthropometry by allocation group and child sex at 8·5 years

	**Control group**	**Intervention group**	**Unadjusted difference (95% CI)**	Multivariable regression*(95% CI)	**Multivariable regression restricted to children without major or chronic illness (95%CI)**
**Weight (kg)**					
Girls	19·55 (2·96)	19·71 (3·15)	0·16 (−0·44 to 0·75)	0·51 (−0·03 to 1·04)	0·51 (−0·03 to 1·06)
Boys	20·53 (3·57)	20·53 (3·49)	−0·00 (−0·67 to 0·66)	0·101 (−0·43 to 0·63)	0·01 (−0·51 to 0·53)
**Height, standing (cm)**					
Girls	120·40 (5·92)	119·98 (5·93)	−0·42 (−1·57 to 0·74)	0·33 (−0·65 to 1·32)	0·44 (−0·51 to 1·39)
Boys	121·05 (5·89)	121·38 (6·12)	0·33 (−0·80 to 1·47)	0·36 (−0·55 to 1·27)	0·14 (−0·78 to 1·06)
**Weight-for-age *Z* score**					
Girls	−2·14 (0·92)	−2·02 (1·02)	0·12 (−0·07 to 0·31)	0·17 (−0·01 to 0·35)	0·17 (−0·01 to 0·35)
Boys	−2·02 (1·09)	−2·04 (1·09)	−0·02 (−0·23 to 0·18)	0·03 (−0·15 to 0·21)	−0·02 (−0·19 to 0·16)
**Height-for-age *Z* score**					
Girls	−1·55 (0·91)	−1·53 (0·93)	0·02 (−0·16 to 0·20)	0·06 (−0·11 to 0·23)	0·08 (−0·08 to 0·24)
Boys	−1·47 (0·93)	−1·44 (0·98)	0·03 (−0·15 to 0·21)	0·06 (−0·10 to 0·22)	0·02 (−0·14 to 0·18)
**BMI-for-age *Z* score**					
Girls	−1·73 (0·87)	−1·58 (0·97)	0·15 (−0·03 to 0·33)	0·18 (0·00 to 0·36)	0·16 (−0·02 to 0·34)
Boys	−1·61 (1·04)	−1·67 (0·99)	−0·06 (−0·25 to 0·13)	−0·02 (−0·20 to 0·16)	−0·05 (−0·23 to 0·13)

Data are mean (SD) unless otherwise stated.

* Multivariable regression models included variables describing air pollution, dietary diversity, food security, maternal education and height, household asset score and residence, by use of robust standard errors. Age was included if not intrinsic to *Z* score.

We noted no difference between allocation groups in direct effects of antenatal multiple micronutrient supplementation, not mediated by birth-size, on *Z* score: 0·04 (95% CI –0·08 to 0·17) for weight-for-age, 0·06 (–0·06 to 0·17) for height-for-age, and 0·05 (–0·08 to 0·18) for BMI-for-age. The appendix shows conditional relative growth results. Generally, there was a tendency for positive effect sizes up to 2·5 years and negative effect sizes up to 8·5 years on both weight-for-age and height-for-age, but these did not reach statistical significance.

Overall, the population was food secure and children had a diverse diet. In the intervention group, 9% of households were food insecure and in the control group 8%. For households in which there was some food insecurity in terms of access, the median HFIAS score was six of 27 in the control group and eight of 27 in the intervention group. The median dietary diversity score was nine of 12 during 7 days in both intervention and control groups. The mean air pollution 24 h time-weighted average (particle mass <4 μm) was 167·2 μg/L (SD 25·2) in the control group and 168·5 μg/L (26·6) in the intervention group.

## Discussion

At follow-up at 8·5 years, we recorded no differences in anthropometric outcomes (with or without adjustment for birth size), conditional relative growth, or blood pressure between groups whose mothers were allocated antenatally to either multiple micronutrient or iron and folic acid supplements. Only head circumference differed in multivariable analysis, to a similar degree as at 2·5 years, but we are cautious about interpreting this finding as one outcome among many.

We can be confident of the findings because TEM and TEM% values were below consensual norms (TEM <3 mm for height and <2 mm for body circumferences and TEM% <1% [<5% for skinfold thicknesses]).^44^, ^45^ Because of balanced allocation, the primary analysis was unadjusted. We developed adjusted models from a conceptual diagram with uncertain assumptions because of the complexity of childhood growth as an outcome. We used causal diagram analysis to make these assumptions explicit, and attempted to adjust for important variables, without overadjustment. The references used to calculate anthropometric *Z* scores differed from those used for children younger than 5 years in the trial and previous follow-up.^39^ This might have led to inconsistencies, but the WHO reference ranges for older children are based on statistical extrapolation from the younger ranges and were designed for compatibility.

We assume that the previous follow-up findings showed a true difference between intervention and control groups in early life (the trial was of high quality and the results in keeping with similar trials). Our interpretation of the findings is that either the differences between groups had disappeared by mid-childhood or that they were not manifest in our indicators. Five other trials of antenatal UNIMMAP supplements have followed up cohorts into childhood (panel). Three groups have assessed child growth,^47^, ^48^, ^49^, ^50^ three have done cognitive tests,^56^, ^57^, ^58^ and one has assessed mortality.^46^ Overall, results of follow-up studies have not shown a lasting difference in anthropometry in children born to mothers taking antenatal multiple micronutrient supplements. In Burkina Faso, Roberfroid and colleagues^49^ recorded increased length-for-age and weight-for-age after 1 year, but the difference had disappeared by 2·5 years. In China, Wang and colleagues^50^ found no difference in wasting, stunting, and underweight at 30 months of age. In Bangladesh, Khan and colleagues^47^, ^48^ reported no difference in weight or body composition at 54 months of age, but reported lower linear growth in children born to mothers in the iron and folic acid control group.

Panel **Research in context**

**Systematic review**

We did a systematic review of follow-up studies of antenatal multiple micronutrient supplementation in low-income and middle-income countries. We searched for randomised controlled trials, with no date restrictions, published in English in Medline, Embase, and PsychINFO with the search terms “micronutrient”, “multiple micronutrient”, or “UNIMMAP” and “pregnancy”, “antenatal”, or “prenatal”. This search yielded 136 records from which we identified 12 relevant trials of multiple micronutrients. We found ten follow-up studies from these trials. One study looked only at mortality and found no difference in overall mortality up to 2 years of age.^46^ Three trials had follow-up studies investigating long-term anthropometry and cardiovascular outcomes. 47, 48, 49, 50, 51 Additionally, two trials of similar antenatal multiple micronutrient combinations to the UNICEF, WHO, United Nations University international multiple micronutrient preparation (UNIMMAP) were followed up for anthropometry and cardiovascular outcomes.^52^, ^53^, ^54^, ^55^ Overall, results of follow-up studies showed no difference in anthropometry in children born to mothers who took multiple micronutrients antenatally compared with those born to mothers who took iron and folic acid. In Bangladesh,^51^ use of multiple micronutrients was associated with a small increase in diastolic blood pressure at 4·5 years of age 0·87 mm Hg (95% CI 0·18–1·56) compared with a control sample that received 30 mg iron and 400 μg folate. There was no difference in systolic blood pressure. In the follow-up from Sarlahi, Nepal, there was no difference in any of the cardiovascular outcomes measured.^54^

**Interpretation**

Overall, findings of studies have shown an increase in birthweight but no consistent lasting effects on anthropometry or cardiovascular outcomes. Our study had the greatest increase in birthweight and, by contrast with the others, was the only one to show to show an increase in weight at 2 years combined with a lower systolic blood pressure. Although our results do not rule out future improvements, combined with those from other trials, they do not suggest a long-term benefit on anthropometry and cardiovascular outcomes.

If the effects of antenatal micronutrient supplementation are transient, they might have been vitiated by childhood in a challenging environment. What this means mechanistically is unclear. Periconceptual and intrauterine development, followed by a time in early childhood of unknown duration, provide an opportunity for epigenetic pattern setting, most commonly via methylation at the CpG dinucleotide site.^3^ Presumably, either methylation is reversible or any changes happened too late to modify growth trajectory if epigenetic mechanisms caused the initial differences.

The second possibility is that long-term effects were present but undetected. Multivariable models showed a difference in mean weight of 295 g, similar to the difference at 2·5 years of age, but a much smaller proportion of the weight of a child aged 8 years. To be able to detect a difference of this magnitude would require a sample roughly ten times as large, and we are unable to say whether or not it was true. A true difference of 300 g would have been equivalent to roughly 1·5% of the cohort mean weight. This amount of difference could have clinical implications, especially in a population such as ours in which many children were undernourished. Perhaps anthropometric differences will emerge at subsequent follow-up, and the present ages of cohorts across all the trials are insufficient to see an effect. Children have low growth rates between the age of 7 and 9 years and the groups might diverge again in adolescence. There is some evidence for this divergence from a study in baboons, in which an effect of changing weaning diet was manifest in females only in adolescence.^59^

If weight is too crude a marker of long-term change, body composition can be more useful. However, neither bioelectrical impedance analysis nor skinfold thickness showed a difference between allocation groups, which suggests no global difference in either lean or fat mass or distribution.

We recorded an increased effect of antenatal micronutrient supplementation at birth in girls, and a differential effect might be biologically plausible (eg, through sex-specific DNA methylation),^60^, ^61^, ^62^ but we found no interaction of child sex and anthropometric indices at follow-up. Other trials have been inconclusive. Friis and colleagues^63^ reported a marginally significant interaction with length, Stewart and colleagues^52^ recorded a non-significant difference in height-for-age *Z* scores in which boys were taller than girls, and Roberfroid and coworkers found no interaction.^64^

Malnutrition was high. Estimates for the Terai from the 2011 Nepal Demographic and Health Survey suggest that 37% of children under 5 were stunted, 11% wasted, and 30% underweight.^65^ We found a 23% higher estimate of low BMI as an index of underweight. The fact that our children were older raises concerns about further deterioration in nutritional status beyond the age of 5 years. Although growth failure is greatest in early life, there seems to be some scope for catch-up in height.

Weight-for-age and levels of obesity in terms of BMI were low, but fat percentage was relatively high. When we applied reference equations for UK children,^66^ the children in our cohort had high fat mass (*Z* score for fat mass −1·66 and −1·92 for lean mass). This finding is in keeping with the so-called thin–fat phenotype common in the region.^67^, ^68^ The effect was determined by relatively high fat mass in boys, with no obvious difference in girls. We noted no difference between the trial groups in morbidity rates. On the basis of parental recall, the data were potentially prone to bias and we were unable to corroborate them reliably with medical records. However, we would expect recall bias to have been similar in both trial groups. The 2·5 mm Hg difference in blood pressure seen at 2·5 years was not sustained at 8·5 years, and there was no difference in kidney dimensions.^69^

Generalisation of the findings might be restricted by non-representative recruitment to the original trial or differential loss to follow-up. Women who chose to participate might have differed systematically from those who did not. Although we adjusted internally for socioeconomic status, rural participants might have been from more affluent groups who could afford to travel to the urban hospital for antenatal care.

So far, meta-analyses have not resulted in a recommendation on the basis of benefits other than increased birthweight.^16^, ^17^, ^18^, ^19^, ^20^ Our supplements began at a minimum 12 weeks' gestation and we await the results of trials investigating periconceptional supplementation. Neither transgenerational nor current environmental influences are likely to be redressed over the course of two trimesters of micronutrient supplementation, but the birthweight advantage might have longer-term health effects. For example, the slower growth of children born in the intervention group compared with children in the control group might be associated with physiological differences that might affect later non-communicable disease.^43^ Further research will undoubtedly add to our understanding of gene–environment interactions, but needs to be augmented by cohort follow-up.
