# Supplementary material for: Effect of antenatal multiple micronutrient supplementation on anthropometry and blood pressure in mid-childhood in Nepal: follow-up of a double-blind randomised controlled trial
Source: Lancet Glob Health. 2014 Nov;2(11):e654–63. doi: 10.1016/S2214-109X(14)70314-6 (PMC4224012; doi:10.1016/S2214-109X(14)70314-6)
Supplement: Supplementary appendix [file mmc1.pdf]

THE LANCET Global Health

Supplementary appendix

This appendix formed part of the original submission and has been peer reviewed. We post it as supplied by the authors.

Supplement to: Devakumar D, Chaube SS, Wells JCK, et al. Effect of antenatal multiple micronutrient supplementation on anthropometry and blood pressure in mid-childhood in Nepal: follow-up of a double-blind randomised controlled trial. *Lancet Glob Health* 2014; **2:** e654–63.

Supplementary Appendix

Antenatal multiple micronutrient supplementation does not result in a difference in anthropometry and blood pressure in mid-childhood: follow-up of a double-blind randomized controlled trial in Nepal

Devakumar D, Chaube SS, Wells JCK, Saville NM, Ayres JG, Manandhar DS, Costello A, Osrin D

Contents

Supplementary table 1: Conditional growth: effect of antenatal multiple micronutrient supplements on growth at different time points 2

Supplementary table 2: Self-reported illness over the last 12 months, by allocation group 2

Supplementary Figure 1: Conceptual model 3

Supplementary Figure 2: Mortality in five periods of follow-up, by allocation 4

	2·5 years		8·5 years	
	Unadjusted difference (95% CI)	Multivariable regression * (95% CI)	Unadjusted difference (95% CI)	Multivariable regression * (95% CI)
**Weight-for-age**	0·08 (-0·06 to 0·22)	0·09 (-0·05 to 0·24)	-0·06 (-0·20 to 0·08)	-0·04 (-0·18 to 0·09)
**Height-for-age**	0·05 (-0·10 to 0·18)	0·08 (-0·04 to 0·21)	-0·10 (-0·24 to 0·04)	-0·09 (-0·12 to 0·04)

Supplementary table 1: Conditional growth: effect of antenatal multiple micronutrient supplements on growth at different time points

* Multivariable regression models included variables describing air pollution, dietary diversity, food security, maternal education and height, household asset score and residence, using robust standard errors.

	Control		Intervention	
**Illness**	**Frequency (%)**	**Median episodes**	**Frequency (%)**	**Median episodes**
**Fever**	346 (82·0)	2	336 (80·2)	2
**Persistent diarrhoea***	2 (0·5)	1	4 (1·0)	2
**Blood in stools**	11 (2·6)	1	19 (4·5)	2
**Pneumonia**	9 (2·1)	1	10 (2·4)	2
**Fast breathing**	7 (1·7)	2	9 (2·2)	2
**Chest indrawing**	8 (1·9)	1·5	7 (1·7)	2

Supplementary table 2: Self-reported illness over the last 12 months, by allocation group

*Diarrhoea lasting for >13 days.

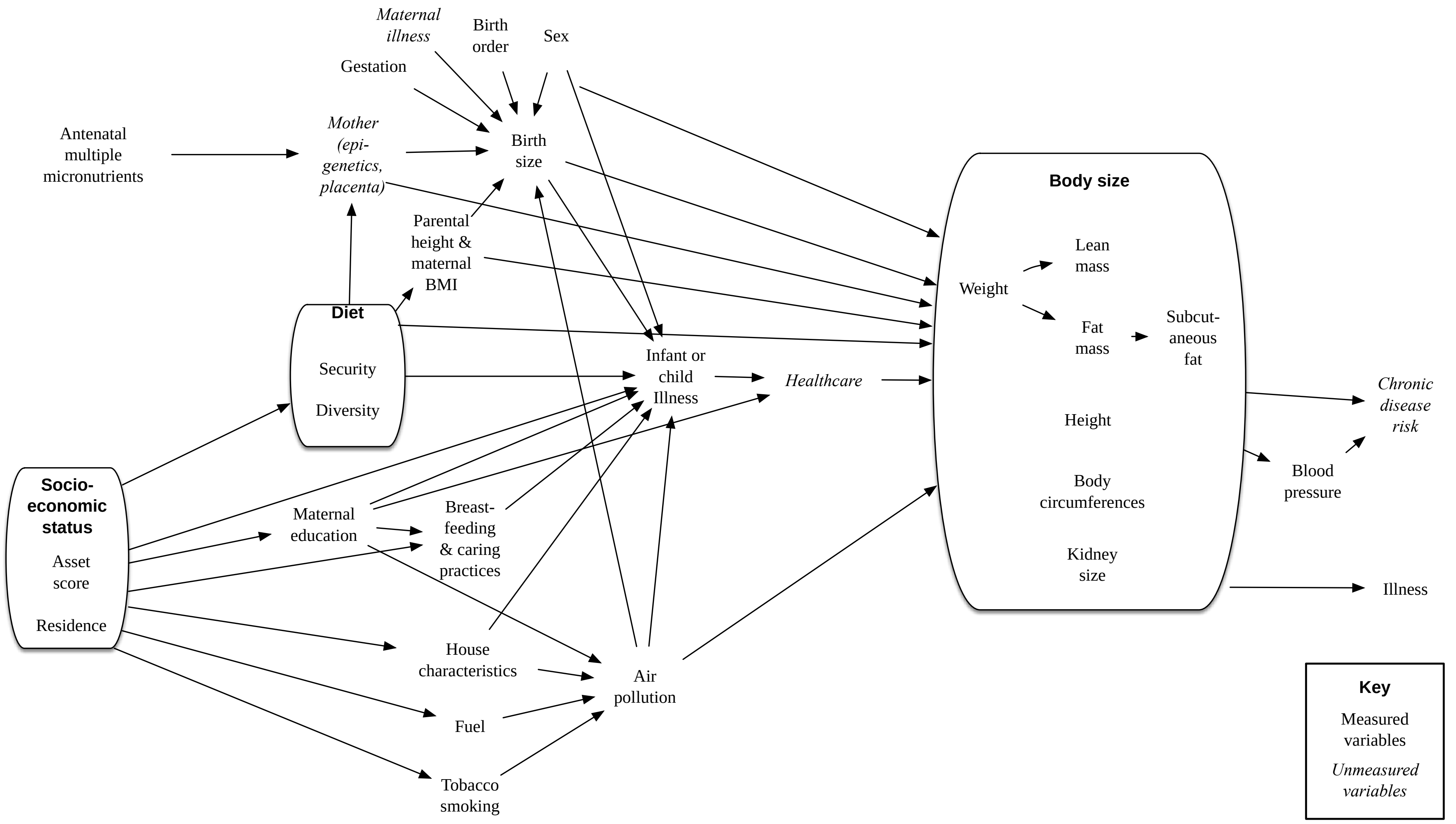

Supplementary Figure 1: Conceptual model

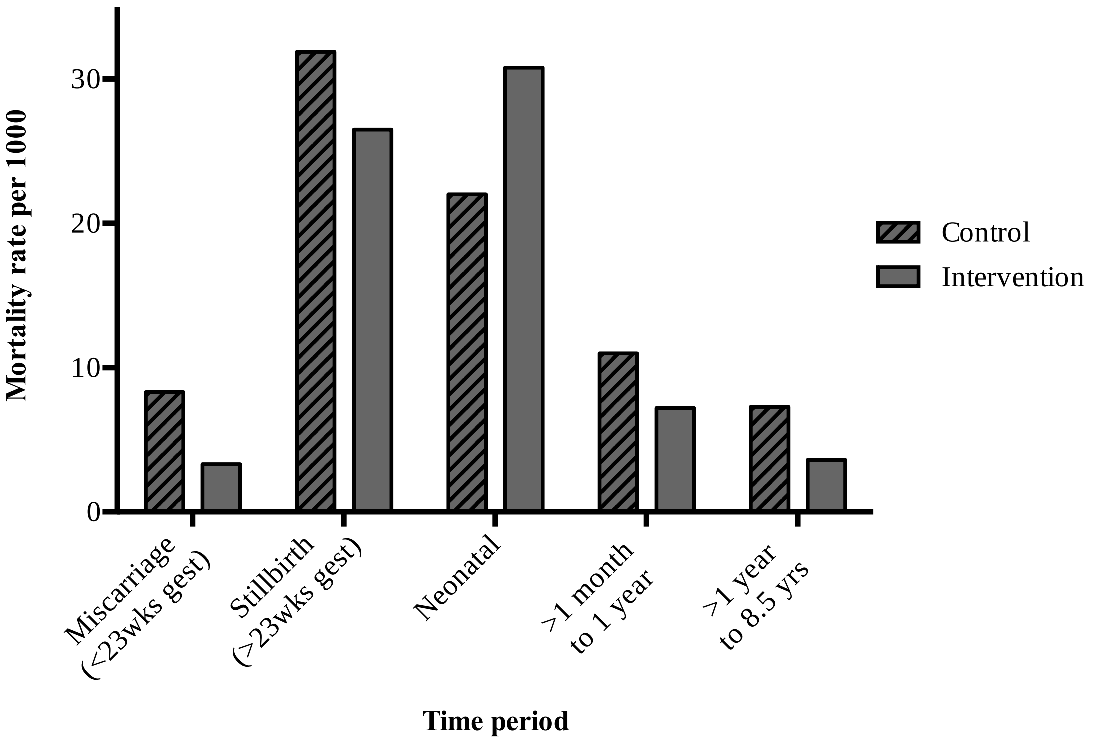

Supplementary Figure 2: Mortality in five periods of follow-up, by allocation
